# Epidemic Dysentery

**Published:** 1853-03

**Authors:** J. B. Evans

**Affiliations:** Frankfort, Ross Co., Ohio


ART. III. *Epidemic Dysentery, by* J. B. Evans, M. D., *Frankfort, Ross Co., Ohio.*

From some unknown cause, epidemics locate themselves in certain districts, when contiguous ones are comparatively healthy.

In this village, and in the neighborhoods to the south and west, we suffered a severe epidemic dysentery the past season, commencing early in July and ending with the approach of winter. A portion of this district is in a malarious locality; but a great proportion of it is hill land and not subject to that influence. But in neither did malaria seem to have any control over the disease.

The disorder was virulent and fatal, giving but little heed to age or sex, but was more fatal with children of from one to three years old. During the prevalence of the epidemic, the weather was unusually cool for the season, and every new accession of coldness seemed to renew and increase its severity. The depressing influence in the disease was very strong, with a typhoid tendency.

The stomach in most bad cases sympathised, and we had to contend with severe and protracted enemies. The retching was very frequent, and sometimes unabated for two or three days together. The ejections were usually small, not exceeding from a tea to a table spoonful at a time of whatever the stomach contained. This was hard to control; and, when added to an almost constant griping, it will readily appear how so many cases proved fatal.

The usual discharge from the bowels was bloody mucus, and in many cases with a preponderance of blood, so as almost to amount to a hemorrhage. Hence, we had to contend with extreme prostration, and that putrid typhoid condition which every practitioner would wish to avoid. A profuse discharge of a serous appearance would take place. This I at first regarded as unfavorable, but it was not the case. And in families where there were a number of cases, it was not unusual for the nurse to say to me that the patient will soon be better, for he has had that beef-brine-looking stool: and I generally found it to be the case. No doubt this was by disgorging the lower intestines of the excessive congestion in their coats. During the prevalence of the flux, but few entirely escaped its influence, and affairs were distressing in the extreme. I am sorry there was no autopsy had. I intended to make such an examination, but a severe attack in my own person prevented me.

I will make a few remarks on the treatment; but the chief point I wish to press is, nourishing the patient in the course of the disease.

Bleeding was not admissable, for the symptoms were typhoid from the onset.

Emetics would sometimes control the vomiting stage, and prove very beneficial.

Blistering and cupping, with the use of sinapisms, were useful in removing local congestion. But blistering had to be used with much care on children, owing to the putrid tendency of the disease. Fomentations over the stomach and bowels did much to alleviate the patient's suffering.

Cathartics were used only with the greatest care. A mild mercurial course sometimes did good. But the mildest laxatives in general could be used, and even these with care. In many, no cathartics of any kind were used, and the cases did well. Here one thing is worthy of a remark. Epsom salts, if used, almost certainly brought on the vomiting stage, which, with care on the part of the medical attendant, could generally be avoided. I know that this opinion of the use of epsom salts in the treatment of dysentery, is in conflict with the generally expressed opinion; but in the epidemic of which I speak, it was the case.

After all is said, opium is the boon. I say *Opium.* It cannot be pressed too strongly. Sulphate of morphia seemed to be the best form. Without it the suffering of the patient was insupportable; he would die of actual pain. Providence is kind in giving us such a cordial for our sufferings. In many cases the amount of morphine used was very great. In my own case, my attending friends gave me within a pittance of a dream, in four weeks; the nervous irritability was so great that the strictest quiet, and the constantly soothing effect of opium was the only alternative. In these extremely nervous cases all treatment was useless, unless the strictest quiet was observed. Too many persons are allowed in the sick room. We should not let delicacy towards the friends of our patients interfere with our duty in this matter; for we are accountable for his life, so far as he is within the reach of means in our power.

*Nourishment.* The duration of an epidemic attack, is usually beyond the starving point, and on this account, if no other, would arise the necessity for food. The starving plan of many of our professional brethren, I hope will be abandoned. Our diseases are becoming more grave and protracted, as the country becomes older and more densely inhabited. I have seen nothing more marked than the good effect derived from food in our scourge of dysentery. Nourishment was necessary from the commencement to the close of an attack. Even when the patient had no relish, and his feelings forbade him eating, he would be much better and suffer less after taking suitable nourishment. In many of the worst cases, the nervous system was completely unstrung, and having a constant griping, the horror produced is indescribable, except to the sufferer, who has felt it in his own person. The suffering was little short of a *fundamental* fire. In short, opium, quiet, and a judicious nourishment were the great essentials in our epidemic. It was necessary to persist in a well selected and oft repeated nourishment, without which the fabric would fall, and I am satisfied that by its neglect many did die.

